# Le syndrome néphrotique idiopathique (SNI) de l’enfant à Dakar: à propos de 40 cas

**DOI:** 10.11604/pamj.2017.26.161.10130

**Published:** 2017-03-21

**Authors:** Younoussa Keita, Ahmed Tall Lemrabott, Assane Sylla, Babacar Niang, El Hadji Fary Ka, Chérif Mohamed Dial, Aliou Abdoulaye Ndongo, Amadou Sow, Claude Moreira, Abdou Niang, Ousmane Ndiaye, Boucar Diouf, Mouhamadou Guélaye Sall

**Affiliations:** 1 Service de Pédiatrie CHU A Le Dantec, Dakar, Sénégal; 2 Service de Néphrologie CHU A, Le Dantec, Dakar, Sénégal; 3 Service d’Anatomopathologie de l’Hôpital Grand Yoff, Dakar, Sénégal; 4 Service de Pédiatrie de l’Hôpital d’Enfants Albert Royer CHU Fann, Dakar, Sénégal; 5 Service de Pédiatrie de l’Hôpital Abass Ndao, Dakar, Sénégal

**Keywords:** Néphrose, corticosensibilité, enfant, Sénégal, Nephrosis, corticosensitivity, child, Senegal

## Abstract

**Introduction:**

L’objectif de ce travail était d’analyser les caractéristiques diagnostiques, thérapeutiques et évolutives de l’enfant atteint de néphrose dans un service de pédiatrie de Dakar.

**Méthodes:**

L’étude était réalisée au service de pédiatrie de l’hôpital Aristide Le Dantec. Il s’agissait d’une étude rétrospective sur une période de 03 ans allant du 1^er^ janvier 2012 au 31 décembre 2014. Ont été inclus tous les patients âgés de 02 ans à 12 ans présentant un tableau de Syndrome néphrotique idiopathique.

**Résultats:**

Quarante cas de néphrose étaient colligés soit une prévalence de 23% parmi les néphropathies prises en charge dans le service. L’âge moyen était de 7,11± 3,14 ans. Le syndrome néphrotique était pur chez 72,5% (n=29) des patients. Les œdèmes des membres inférieurs étaient présents chez 100% des patients, l’oligurie dans 55% (n=22) et l’HTA dans 5% (n=2) des cas. La protéinurie moyenne était de 145,05 ± 85,54 mg/kg/24heures. La protidémie moyenne était de 46,42 ±7,88 g/L et l’albuminémie moyenne de 17,90 ± 7,15 g/L. Trente-neuf patients avaient reçu une corticothérapie à base de prednisone. La corticosensibilité était retenue chez 77% (n=30) des patients et la corticorésistance chez 13% (n=5) des cas. Le facteur de mauvaise réponse à la corticothérapie était un niveau de protéinurie initiale supérieure à 150 mg/kg/jour (p = 0,024). La biopsie rénale était réalisée chez 18% (n=7) des patients et retrouvait dans 57,2% (n=4) des cas une hyalinose segmentaire et focale. Le cyclophosphamide et l’azathioprine étaient associés aux corticoïdes dans 10% (n=4) des cas chacun. Le taux de rémission globale était de 89,8%. L’évolution vers l’insuffisance rénale chronique était notée chez trois (03) des patients.

**Conclusion:**

La néphrose représentait près du quart des néphropathies prises en charge dans notre service. Le taux de rémission globale était élevé. Le seul facteur de mauvaise réponse à la corticothérapie était le niveau de protéinurie initiale élevée. En cas d’indication de la biopsie rénale chez nos patients, la HSF était la lésion la plus fréquemment retrouvée.

## Abstract (en)

**Introduction:**

This study aimed to analyze the diagnostic, therapeutic, and evolutionary features of nephrosis in children in a pediatric department in Dakar.

**Methods:**

The study was carried out in the Department of Pediatrics at the Aristide Le Dantec Hospital. We conducted a retrospective study over a period of 3 years from 1 January 2012 to 31 December 2014. All patients aged 2-12 years with idiopathic nephrotic syndrome were included in the study.

**Results:**

Forty cases of nephrosis were collected, that is to say a prevalence of 23% among patients with kidney disease treated in the Department of Pediatrics. The average age was 7.11 ± 3.14 years. 72.5% (n=29) of patients suffered from pure nephrotic syndrome. Lower limb edema was present in 100% of patients, oliguria in 55% (n=22) and high blood pressure (HBP) in 5% (n=2) of cases. Median proteinuria was 145,05 ± 85,54 mg/kg/24 hours. Median protidemia was 46,42 ±7.88 g/L and median albumin was 17.90 ± 7.15 g/L. Thirty nine patients were treated with prednisone-based corticosteroid therapy. Corticosensitivity was retained in 77% (n=30) patients and corticoresistance in 13% (n=5) of cases. The factor of poor response after corticosteroid therapy was initial proteinuria greater than 150 mg/kg/day (p = 0.024). Renal biopsy was performed in 18% (n=7) of patients which showed focal and segmental hyalinosis in 57.2% (n=4). Cyclophosphamide and azathioprine were associated with corticosteroids in 10% (n=4) of cases respectively. The overall remission rate was 89.8%. The evolution toward chronic renal failure was observed in three patients.

**Conclusion:**

Nephrosis accounted for almost one quarter of all cases of kidney disease treated in our Department. It has high overall remission rate. The only factor contributing to poor response after corticosteroid therapy was high levels of initial proteinuria. Focal and segmental hyalinosis was the most frequently found lesion diagnosed by renal biopsy.

## Introduction

La néphrose ou syndrome néphrotique idiopathique (SNI) est la plus fréquente des néphropathies glomérulaires de l’enfant [1, 2]. Elle représente 90% des syndromes néphrotiques (SN) de l’enfant avant 10 ans et 50% après cet âge [3]. Peu d’études font mention d’un suivi à long terme d’enfants africains atteints de SNI [4, 5]. L’objectif de ce travail était d’analyser les caractéristiques diagnostiques, thérapeutiques et évolutives de l’enfant atteint de néphrose au Sénégal.

## Méthodes

L’étude était réalisée au service de pédiatrie de l’hôpital Aristide Le Dantec de Dakar. Il s’agissait d’une étude rétrospective sur une période de 03 ans allant du 1^er^ janvier 2012 au 31 décembre 2014. Les enfants inclus étaient âgés entre 02 et 12 ans et présentaient un SNI. Le SN était défini par l’association d’une protéinurie supérieure ou égale à 50 mg/kg/24h, d’une hypoalbuminémie inférieure à 30g/L et d’une hypoprotidémie inférieure à 60g/L. Le caractère idiopathique du SN était retenu devant un bilan étiologique négatif comportant une recherche de drépanocytose, de lupus, de diabète, de VIH et de VHB. Les patients ont été traités par corticoïde selon le protocole proposé par la Société de Néphrologie Pédiatrique (SNP) [6, 7]. La corticosensibilité était définie par la disparition de la protéinurie à l’issue de quatre semaines de traitement oral à base de prednisone à la dose de 2mg/kg/j ou après 3 bolus de méthylprednisolone à la dose de 1g/1,73m^2^ de surface corporelle par bolus. La corticorésistance était définie par l’absence de rémission huit jours après les perfusions de méthylprednisolone. La corticodépendance était définie par une rechute pendant la décroissance de la corticothérapie ou moins de trois mois après l’arrêt de celle-ci. Nous avions analysé les données diagnostiques, thérapeutiques et évolutives grâce au logiciel SPSS version 18. L’étude descriptive était réalisée par le calcul des fréquences pour les variables qualitatives et par le calcul des moyennes avec leur écart-type pour les variables quantitatives. L’étude analytique était faite avec des tableaux croisés. Les moyennes avec leur écart-type et les pourcentages ont été comparés à l’aide du test de Student et du test du Khi 2, suivant leurs conditions d’applicabilité, avec un seuil de significativité si le « p » était inférieur à 0,05.

## Résultats

**Figure 1:**
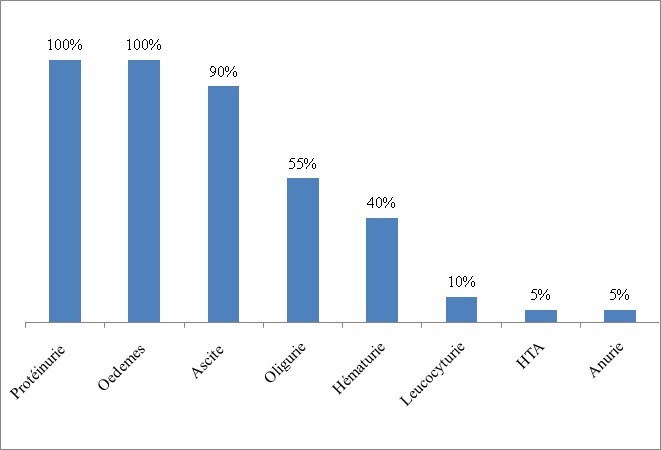
Représentation graphique des manifestations cliniques du syndrome néphrotique idiopathique chez nos patients

**Figure 2:**
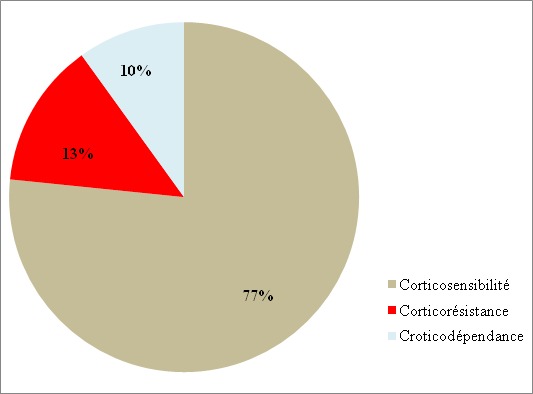
Répartition des patients selon la réponse à la corticothérapie initiale

**Figure 3:**
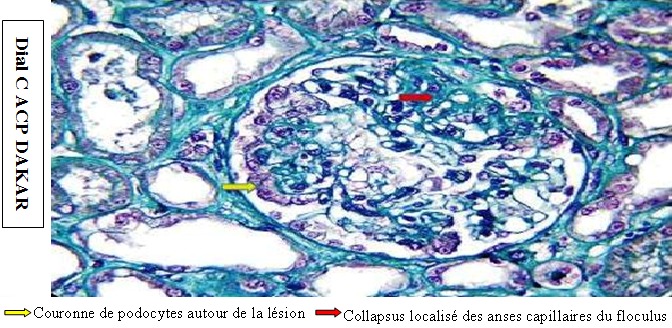
Biopsie rénale montrant une lésion de hyalinose segmentaire et focale chez un enfant présentant un syndrome néphrotique corticorésistant (Trichome de Masson vert lumière G x 200

Parmi 2565 enfants consultés dans le service durant la période d’étude, 6,8% (n=174) avaient une néphropathie. Notre étude concernait 40 patients soit une prévalence hospitalière de 1,5% et une prévalence de 23% parmi les néphropathies prises en charge au service de pédiatrie. L’âge moyen était de 7,11 ± 3,14 ans et le sex-ratio de 1,85. La majorité des patients (55%) provenait de la zone semi-urbaine de Dakar. La consanguinité parentale était retrouvée dans 27,5% (n=11) des cas. Le poids moyen des enfants était de 23,82 ± 10,63 kg et leur taille moyenne de 110,83 ± 18,01 cm. La protéinurie était présente dans 100% des cas à la bandelette urinaire, de même que les œdèmes des membres inférieurs à l’examen physique (Figure 1). A la biologie, la protéinurie moyenne était de 145,05 ± 85,54 mg/kg/24 heures. La protidémie moyenne était de 46,42 ±7,88 g/L et l’albuminémie moyenne de 17,90± 7,15 g/L. L’insuffisance rénale aigue transitoire était notée chez 7,7% (n= 3) des patients. Au compte d’Addis, 27,5% (n=11) des patients avaient une hématurie et 40% (n=16) une leucocyturie. A l’admission, l’examen cytobactériologique des urines était positif chez 7,7% (n=3). Les germes isolés étaient *Escherichia Coli*, *Klebsiella Oxytoca*, et *Klebsiella Pneumoniae*. A l’échographie, la taille des reins était augmentée chez 20 % (n=8) des patients. Dans les autres cas, l’échostructure rénale était normale. Le syndrome néphrotique était pur chez 72,5% (n=29) des patients. Un enfant était décédé dans un tableau d’insuffisance rénale aigue avant le début du traitement. Trente-neuf patients avaient reçu une corticothérapie à base de prednisone. La durée moyenne du traitement d’attaque était de 1,02 ± 0,11 mois. La corticosensibilité était retenue chez 77% (n=30) (Figure 2). Le facteur de mauvaise réponse à la corticothérapie était un niveau de protéinurie initiale supérieure à 150 mg/kg/jour (p = 0,024). Les complications infectieuses bactériennes liées à la corticothérapie au long cours étaient notées chez 15,4% (n=6) dont 03 cas d’infections urinaires, 01 cas de méningite, 01 cas de cellulite et 01 cas de sepsis sévère. Les mycoses superficielles de la peau et des ongles étaient notées chez 72% (n=28) des enfants. La biopsie rénale était réalisée chez 18% (n=7) (Tableau 1). Elle révélait une hyalinose segmentaire et focale (HSF) dans 57,2% (n=4) des cas (Figure 3). Le cyclophosphamide ou l’azathioprine était associé aux corticoïdes à faible dose dans 10% (n=4) des cas chacun. Le taux de rémission par le cyclophosphamide était de 75% (n=03) et de 50% (n=02) par l’azathioprine. Le taux de rémission globale était de 89,8% (n=35). Au cours de l’évolution des cas d’HSF, 75% (n=03) des patients ont évolué vers l’insuffisance rénale chronique.

**Tableau 1:** Principales lésions retrouvées à la biopsie rénale chez nos patients

Lésions retrouvées	Indications de la biopsie rénale		Total (n=7)
	Corticorésistance	Rechutes fréquentes	
HSF	04	-	04 (57,2%)
LGM	-	02	02 (28,5%)
PMD	-	01	01 (14,3%)

HSF: hyalinose segmentaire et focale; LGM: lésions glomérulaires minimes; PMD: prolifération mésangiale diffuse

## Discussion

La fréquence du SNI varie selon les séries. En effet, en Ouganda et au Nigéria, Gbadoé et al. ont rapporté une fréquence de 2%, contre 0,02 à 0,04% aux Etats-Unis, en Chine et au Royaume-Uni [4]. Au Congo Brazzaville, Moyen et al. [8] ont trouvé une fréquence de 0,8%. Dans notre étude, l’âge moyen était de 7,11 ± 3,14 ans. Ce résultat était comparable à celui retrouvé dans l’étude de Gbadoé et al. qui rapportaient un âge moyen entre 6 et 8 ans en Afrique noire, contre 5 ans en Europe, en Asie et en Amérique [4]. L’âge moyen tardif noté dans les pays en voie de développement serait vraisemblablement lié au retard diagnostique. Nous avions noté une prédominance masculine avec un sex-ratio de 1,85. Ce résultat était similaire à celui retrouvé respectivement par Moyen et al. [8] à Brazzaville et Ernould et al [9] en France. De plus, les données épidémiologiques apportent des informations qui permettent de suggérer que le syndrome néphrotique de l’enfant est un modèle de maladie de l’interaction gène-environnement [10]. Au Sénégal, Ndongo et al. avaient noté une incidence plus élevée du SN entre les mois de mars et mai [5]. Sur le plan clinique, les œdèmes de type rénal étaient présents chez tous nos patients (100%). Moyen et al. [8] ont retrouvé dans leur étude le même résultat, de même que Mabiala-Babela et al. [11] au Congo et Bourquia et al. [8] au Maroc, tandis que Safaei [12] a rapporté un résultat à peu près similaire (95%). Cette présence permanente du syndrome œdémateux de type rénal pourrait s’expliquer par le retard à la consultation et/ou au diagnostic. Le SN était pur chez 72,5% de nos patients. La nette prépondérance du SN pur au cours de la néphrose est confirmée par les données de la littérature. En effet, Ernould et al. [9] a retrouvé dans sa série 89% de SN pur. La corticosensibilité était de 77% dans notre série. A Abidjan, Adonis-Koffy et al. rapportaient un taux de corticosensibilité de 84% [13]. Le degré élevé de la protéinurie des 24 heures constituait un facteur de mauvais pronostic sur l’évolution du SN vers la corticodépendance ou la corticorésistance. Par ailleurs, la protéinurie a aussi été reconnue comme facteur prédictif de mortalité cardiovasculaire, indépendamment de la filtration glomérulaire et d’autres facteurs de risque cardiovasculaire [14]. La sur-expression des facteurs de croissance intrarénaux occupe une place centrale dans la progression des lésions rénales. Cette sur-expression pourrait être secondaire à la surcharge tubulaire en protéines [15, 16]. La ponction biopsie rénale n’est pas indiquée en cas de SNI de l’enfant mais dans certaines situations comme la corticorésistance, la biopsie peut être réalisée. Dans notre étude, la PBR a été pratiquée chez 18% des patients. Au Congo Brazzaville, la PBR était faite chez 19,4% des patients dans l’étude de Moyen et al. [8], tandis que Mabiala-Babela et al. [11] rapportaient 25,24% de biopsie rénale pratiquée dans sa série. Quant à Bourquia et al. [17], ils rapportaient 84,61% de biopsie rénale au Maroc. Cette disparité dans la fréquence des biopsies rénales dans les différentes séries pourrait s’expliquer par la variabilité des indications et des méthodologies d’étude. Dans notre série, la HSF était la lésion histologique la plus fréquemment retrouvée en cas d’indication de la PBR au cours SNI. En Afrique, Il ressort de plusieurs études, la fréquence élevée de la HSF en cas d’indication de la biopsie rénale chez l’enfant. Ainsi, au Ghana, les auteurs rapportaient une fréquence de HSF de 77% [18], 34,8% au Congo Brazzaville [8] et 25% au Maroc [17].

## Conclusion

La néphrose représente près d’un quart des néphropathies prises en charge dans notre service. La corticosensibilité était élevée et la corticorésistance faible. Le seul facteur de mauvaise réponse à la corticothérapie est le niveau de protéinurie initiale élevée. L’utilisation des immunosuppresseurs associés aux corticoïdes à faible dose avait permis d’améliorer le niveau de rémission globale. Dans les cas où la biopsie rénale était indiquée chez nos patients, la HSF était la lésion la plus fréquemment retrouvée.

### Etat des connaissances actuelles sur le sujet

- Il s’agit d’une maladie ubiquitaire qui serait beaucoup plus fréquente en milieu intertropical comparé à la zone tempérée du fait de l’endémicité des infections;
- L’étiologie de la maladie n’est pas connue;
- Le traitement par les corticoïdes (prednisone) au long cours a fait sa preuve depuis les années 60.

### Contribution de notre étude à la connaissance

- L’âge moyen de découverte dans notre étude est tardif par rapport à celui dans les pays développés;
- La corticosensibilité est élevée chez l’enfant sénégalais;
- En cas d’indication de la biopsie rénale pour corticorésistance, la hyalinose segmentaire et focale (HSF) est plus fréquente que les autres lésions histologiques.
